# Tamoxifen therapy benefit predictive signature coupled with prognostic signature of post-operative recurrent risk for early stage ER+ breast cancer

**DOI:** 10.18632/oncotarget.6260

**Published:** 2015-10-30

**Authors:** Hao Cai, Xiangyu Li, Jing Li, Lu Ao, Haidan Yan, Mengsha Tong, Qingzhou Guan, Mengyao Li, Zheng Guo

**Affiliations:** ^1^ Department of Bioinformatics, Key Laboratory of Ministry of Education for Gastrointestinal Cancer, Fujian Medical University, Fuzhou, China; ^2^ College of Bioinformatics Science and Technology, Harbin Medical University, Harbin, China

**Keywords:** breast cancer, relative expression ordering, prognostic signature, predictive signature, tamoxifen

## Abstract

Two types of prognostic signatures for predicting recurrent risk of ER+ breast cancer patients have been developed: one type for patients accepting surgery only and another type for patients receiving post-operative tamoxifen therapy. However, the first type of signature cannot distinguish high-risk patients who cannot benefit from tamoxifen therapy, while the second type of signature cannot identify patients who will be at low risk of recurrence even if they accept surgery only. In this study, we proposed to develop two coupled signatures to solve these problems based on within-sample relative expression orderings (REOs) of gene pairs. Firstly, we identified a prognostic signature of post-operative recurrent risk using 544 samples of ER+ breast cancer patients accepting surgery only. Then, applying this drug-free signature to 840 samples of patients receiving post-operative tamoxifen therapy, we recognized 553 samples of patients who would have been at high risk of recurrence if they had accepted surgery only and used these samples to develop a tamoxifen therapy benefit predictive signature. The two coupled signatures were validated in independent data. The signatures developed in this study are robust against experimental batch effects and applicable at the individual levels, which can facilitate the clinical decision of tamoxifen therapy.

## INTRODUCTION

Breast cancer is the most prevalent cancer among women worldwide and approximately 70% of cases express estrogen receptor [1, 2]. Tamoxifen has been the major adjuvant therapy for ER+ breast cancer, but one-third of early-stage patients treated with tamoxifen after surgery for five years will experience a relapse of cancer within fifteen years [3, 4]. To reduce the recurrent rate, the majority of early-stage ER+ patients also receive adjuvant chemotherapy after surgery, of which only a small proportion will ultimately benefit from the adjuvant chemotherapy, while all remain at risk of toxic side-effects [5]. Therefore, a signature for identifying patients who can benefit from tamoxifen therapy is required. In addition, although continuing tamoxifen therapy has been found to produce a reduction in recurrence and mortality for ER+ breast cancer [6], the patients treated with tamoxifen for a long time may suffer from side-effects, such as deep-vein thrombosis, endometrial cancer, pulmonary embolus, bone loss, stroke and genito-urinary system dysfunction [7–9]. If patients at low risk after surgery can be discriminated from patients at low risk with the help of tamoxifen therapy, clinicians could make proper decisions on tamoxifen therapy for the two distinct groups to assure its effectiveness and minimize adverse treatment effects.

Many prognostic signatures, such as the 70-gene signature reported by van‘t Veer *et al.* [10] and the 76-gene signature reported by Wang *et al.* [11], have been developed for predicting clinical outcome of ER+ breast cancer patients accepting surgery only [12, 13]. Although these drug-free prognostic signatures could be used to guide the recommendation of adjuvant tamoxifen therapy based on the finding that only patients in the high-risk group may benefit from tamoxifen therapy [14], they cannot further distinguish high-risk patients who cannot benefit from tamoxifen therapy. Some other researchers used samples of ER+ breast cancer patients receiving post-operative tamoxifen therapy to develop signatures for predicting clinical outcome of these patients [15–18]. Patients with low risk of recurrence recognized by such signatures are considered to be able to benefit from tamoxifen therapy and might be recommended to tamoxifen therapy. However, some of these patients will be at low risk of recurrence if they accept surgery only and actually need no tamoxifen therapy after surgery. Obviously, these problems need to be solved.

Most of previously reported signatures are based on risk scores, usually calculated as some summaries of expression measurements of the signature genes, to allocate patients into different prognostic groups [10–12, 16–18]. However, such risk-score based signatures often fail in independent samples [19–22] because risk scores summarized from expression measurements of signature genes are sensitive to experimental batch effects [22–24]. As the applications of such risk-score based signatures require data normalization using a set of samples [10–12, 16–18], the risk classification of a sample depends on the risk composition of the samples analyzed together with this sample [25]. In contrast, the signatures based on the within-sample relative expression orderings (REOs) of gene pairs are insensitive to experimental batch effects and invariable to monotonic data normalization [22–25]. Based on this unique advantage, the REO-type prognostic signatures can perform robustly in inter-laboratory datasets and allow application at the individual levels [15, 26]. Another important advantage of REOs is that we can pool samples from different small datasets together for further analysis, which is of special interest given that the discovery and validation of prognostic signatures often need a large number of samples [26]. However, one major problem of finding REO-type prognostic signatures is that the number of gene pairs constituted by all genes in a dataset is extremely large, leading to a super-high dimensional problem and consequently a over-fitting problem [27]. To improve the robustness of analytical results, a commonly approach is to start with pathway analyses to develop a signature based on the phenomenon that signatures identified from different samples are often closely related in functions [28, 29]. Our previous research has found that within-sample REOs are overall stable in particular types of normal human tissue but widely disturbed in the corresponding cancers, which could provide the basis for pathway analysis based on REOs [30].

## RESULTS

### Drug-free prognostic signature of post-operative recurrent risk

Using the gene expression profiles of 167 normal breast tissue samples measured by the GPL96 platform (Affymetrix HG-U133A) (Table 1), we identified 22,717,681 stable gene pairs, each of which had a stable REO in more than 99% of normal samples. Similarly, we identified 45,603,713 stable gene pairs in 407 normal breast tissue samples (Table 1) measured by the GPL570 platform (Affymetrix HG-U133 plus 2.0). The two lists of stable gene pairs had 17,507,393 overlaps, of which more than 98% had identical REOs, which was highly unlikely to occur by chance (*p* < 1.0E–16, binomial distribution test, see *Methods*). The highly stable REOs reflect the coordinated structure of gene expressions in the normal breast tissue, based on which we could characterize every cancer sample by identifying gene pairs with reversal REOs in this sample [30]. In the following text, we used the gene pairs with stable normal REOs consistently detected by both the GPL96 and GPL570 platforms to characterize cancer samples.

**Table 1:** Description of normal breast tissue datasets and ER+ breast cancer tissue datasets used in this study

	GEO Acc	Platforms	Number of normal^a^	Number of cancer
Samples of normal breast tissue	GSE15852 [31]	GPL96	43	
	GSE20437 [32]	GPL96	42	
	GSE21947 [33]	GPL96	30	
	GSE9574 [34]	GPL96	29	
	GSE16873 [35]	GPL96	12	
	GSE48984 [36]	GPL96	6	
	GSE6883 [37]	GPL96	3	
	GSE6596 [38]	GPL96	2	
	GSE10780 [39]	GPL570	143	
	GSE26457 [40]	GPL570	113	
	GSE30010	GPL570	107	
	GSE10810 [41]	GPL570	27	
	GSE42568 [42]	GPL570	17	
Samples of patients accepting surgery only	**GSE7390** [43]	GPL96		134
	**GSE6532_ut^b^** [44]	GPL96		85
	GSE2034 [11]	GPL96		209
	GSE4922_ut^c^ [45]	GPL96		116
Samples of patients receiving post-operative tamoxifen therapy	**GSE17705** [16]	GPL96		298
	**GSE12093** [14]	GPL96		136
	**GSE6532_tt1^b^** [44]	GPL570		87
	GSE6532_tt2^b^ [44]	GPL96		176
	GSE4922_tt^c^ [45]	GPL96		66
	GSE9195 [46]	GPL570		77

Note:

a To determine stable gene pairs in normal tissue, from each dataset only normal samples were collected and the information of disease samples was not presented.

b GSE6532 series contains three type samples: GSE6532_ut, samples of the lymph-node-negative patients accepting surgery alone; GSE6532_tt1, samples of the patients receiving post-operative tamoxifen therapy measured by GPL570 platform; GSE6532_tt2, samples of the patients receiving post-operative tamoxifen therapy measured by GPL96 platform.

c GSE4922 series contains two type samples: GSE4922_ut, samples of the lymph-node-negative patients accepting surgery alone; GSE4922_tt, samples of the patients receiving post-operative tamoxifen therapy. The datasets in bold were discovery cohort.

The 219 samples of lymph-node-negative patients accepting surgery only, collected from the GSE7390 and GSE6532_ut datasets (Table 1), were used as the discovery cohort to develop a drug-free prognostic signature of post-operative recurrent risk. Firstly, based on the 1320 canonical pathways documented in the C2 collection of the MSigDB, we identified pathways whose disrupted REOs were significantly associated with recurrence-free survival (RFS). Here, RFS was used in a broad sense to represent the prognostic end points of both local recurrence and distant recurrence [47]. For each pathway, among the intra-pathway gene pairs with stable REOs in normal tissue, the frequency of gene pairs with reversal REOs in each cancer sample was calculated, termed as the disruption index of this pathway in this sample. Then, using the univariate Cox proportional-hazard model, with FDR < 5%, we identified 37 pathways whose disruption indexes were significantly correlated with RFS (Supplementary Table 1). To search for significantly correlated RFS-relevant pathways, we evaluated the correlations of the disruption indexes among the RFS-relevant pathways using Spearman rank correlation test with FDR < 5%. After linking every two significantly correlated pathways whose Spearman rank correlation coefficient was larger than 0.6, we found 23 pathways that could be connected together as a large network (Supplementary Figure 1). Many of these 23 pathways are well-known metastasis-associated pathways, including P53 and RAS signaling pathways, cell-cycle-related pathways and immunity-related pathways, as described in Supplementary Table 1. Finally, we searched for prognostic signature of gene pairs within these 23 RFS-relevant pathways, which could be regarded as the core drug-free RFS-relevant pathways. By this way, the number of gene pairs to be searched was greatly reduced, which was expected to be able to improve the robustness of signature selection.

**Figure 1:**
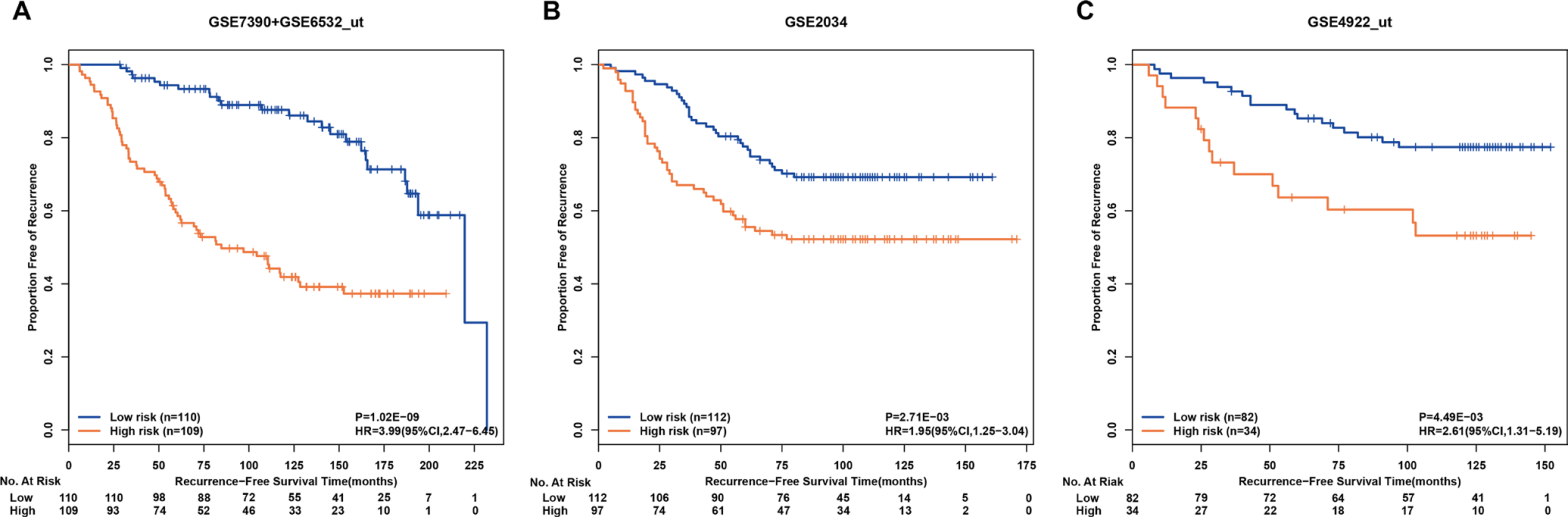
Kaplan-Meier estimates of recurrence-free survival in patients accepting surgery only according to the drug-free prognostic signature of post-operative recurrent risk Recurrence-free survival curves in the discovery cohort (**A**) the first validation cohort (**B**) and the second validation cohort (**C**).

Within the 23 pathways, there were 19,844 gene pairs with stable REOs in the normal breast tissue. From these gene pairs, using the univariate Cox proportional-hazard model, with FDR<10%, we indentified 138 gene pairs whose reversal REOs were significantly correlated with poor RFS (see *Methods*). From these 138 gene pairs, a forward-stepwise selection algorithm was performed to obtain a subset of gene pairs whose C-index reached maximum (see *Methods*) based on the following classification rule: patients with no reversal gene pairs in the subset were assigned to the low-risk group and all the other patients were assigned to the high-risk group. Finally, we extracted nine gene pairs (Table 3), termed as the drug-free prognostic signature of post-operative recurrent risk, which classified the discovery cohort into a low-risk group with 110 patients and a high-risk group with 109 patients. As shown in Figure 1A, the patients in the low-risk group had significantly better RFS than the patients in the high-risk group (HR = 3.99, 95%CI:2.47–6.45, *p* = 1.02E–09, C-index = 0.69).

**Table 2:** Clinical characteristics of patients with ER+ breast canc

	Patients accepting surgery only				Post-operative tamoxifen-treated patients					
	GSE7390	GSE6532_ut	GSE2034	GSE4922_ut	GSE17705	GSE12093*	GSE6532_tt1	GSE6532_tt2	GSE4922_tt	GSE9195
All samples	134	85	209	116	298	136	87	176	66	77
Median follow-up (months)	125.8 (6.1–232)	89.2 (6.1–176.8	86 (2–171)	122 (6–152)	97.8 (6.0–195.2	85.0 (7.6 to 192.6)	138.5 (6.3–205.0)	62.8 (0.3–155.1)	78 (0–152)	99.8 (7–137.5)
**Age**										
Median age	47 (24–60)	55 (32–71)	—	66 (32–90)	—	—	62 (43–86)	65 (40–88)	65.5 (45–88)	65 (42–82)
< = 55	115 (86%)	44 (52%)	—	34 (29%)	—	27 (20%)	14 (16%)	31 (18%)	11 (17%)	16 (21%)
> 55	19 (14%)	41 (48%)	—	82 (71%)	—	109 (80%)	73 (84%)	145 (82%)	55 (83%)	61 (79%)
**Tumor Grade**										
1	29 (22%)	29 (34%)	—	48 (41%)	—	30 (22%)	17 (19.5%)	33 (19%)	11 (17%)	14 (18%)
2	68 (51%)	62 (72%)	—	58 (50%)	—	43 (32%)	37 (43%)	93 (53%)	42 (63%)	20 (26%)
3	35 (26%)	12 (14%)	—	10 (9%)	—	8 (6%)	16 (18%)	27 (15%)	13 (20%)	24 (31%)
NA	2 (1%)	13 (15%)	—	0	—	55 (40%)	17 (19.5%)	23 (13%)	0	19 (25%)
**Tumor Size**										
≤ 2cm	76 (57%)	54 (64%)	—	84 (72%)	—	—	43 (49%)	67 (38%)	18 (27%)	34 (44%)
> 2cm	58 (43%)	31 (36%)	—	32 (87%)	—	—	44 (51%)	109 (62%)	48 (73%)	43 (56%)
**Lymph Node**										
negative	134 (100%)	85 (100%)	209 (100%)	116 (100%)	175 (59%)	—	29 (33%)	85 (48%)	17 (26%)	41 (53%)
positve	0	0	0	0	112 (37%)	—	58 (67%)	82 (47%)	46 (70%)	36 (47%)
NA	0	0		0	11 (4%)	—	0	9 (5%)	3 (4%)	0
**Tumor Stage#**										
T1/2	134 (100%)	85 (100%)	209 (100%)	116 (100%)	—	128 (94%)	87 (100%)	176 (100%)	—	77 (100%)
T3/4	0	0	0	0	—	7 (5%)	0	0	—	0
NA	0	0	0	0	—	1 (1%)	0	0	—	0

* The clinical data of GSE12093 was obtained from the paper and the detail information for each patient was not provided in GEO.

# The tumor stage information of each dataset was from the corresponding reference paper.

**Table 3:** The drug-free prognostic signature of post-operative recurrent risk

	Gene A			Gene B	
Gene ID	Gene Symbol	Gene Full Name	Gene ID	Gene Symbol	Gene Full Name
55182	RNF220	ring finger protein 220	27338	UBE2S	ubiquitin-conjugatig enzyme E2S
6124	RPL4	ribosomal protein L4	3315	HSPB1	heat shock 27kDa protein 1
7327	UBE2G2	ubiquitin-conjugatig enzyme E2G 2	51588	PIAS4	protein inhibitor of activated STAT, 4
22794	CASC3	cancer susceptibility candidate 3	23658	LSM5	LSM5 homolog, U6 small nuclear RNA and mRNA degradation associated
6205	RPS11	ribosomal protein S11	9861	PSMD6	proteasome 26S subunit, non-ATPase 6
896	CCND3	cyclin D3	983	CDK1	cyclin-dependent kinase 1
5689	PSMB1	proteasome subunit beta 1	27338	UBE2S	ubiquitin-conjugatig enzyme E2S
1021	CDK6	cyclin-dependent kinase 6	990	CDC6	cell division cycle 6
5707	PSMD1	proteasome 26S subunit, non-ATPase 1	27338	UBE2S	ubiquitin-conjugatig enzyme E2S

Gene A has a higher expression level than Gene B in normal breast tissues.

In the first independent validation cohort of the GSE2034 dataset, the drug-free prognostic signature identified 112 patients at low risk and 97 patients at high risk, respectively, while the RFS of the former was significantly better than that of the latter (HR = 1.95, 95%CI:1.25–3.04, *p* = 2.71E–03, C-index = 0.59, Figure 1B). The drug-free prognostic signature was also validated in another independent GSE4922_ut dataset: the low-risk group of 82 patients had a significantly better RFS than the high-risk group of 34 patients (HR = 2.61, 95%CI:1.31–5.19, *p* = 4.49E–03, C-index = 0.60, Figure 1C). The first validation cohort lacks clinical data, while multivariate Cox analyses for the discovery cohort and the second validation cohort both showed the drug-free prognostic signature was a strong independent factor for predicting the post-operative recurrent risk after adjusting age, tumor size and histology grade (Table 4).

**Table 4:** Univariate and multivariate Cox regression analysis for the drug-free prognostic signature

	Univariate model		Multivariate model	
Variables	HR (95%CI)	*P*	HR (95%CI)	*P*
The 204 samples of the discovery cohort				
The nine gene pairs	5.22 (3.08–8.86)	9.19E–10	5.10 (2.98–8.72)	2.74e–09
Age (> 55 vs. ≤ 55)	1.17 (0.71–1.92)	0.5473	1.14 (0.68–1.92)	0.6136
Grade (3 vs. 2 vs. 1)	1.24 (0.91–1.69)	0.1731	0.95 (0.69–1.31)	0.7590
Size (> 2 vs. ≤ 2 cm)	2.14 (1.37–3.33)	8.07E–04	1.90(1.19–3.02)	6.99e–03
The 116 samples of the second validation cohort				
The nine gene pairs	2.61 (1.31–5.19)	0.0062	2.16 (1.05–4.46)	0.0362
Age (> 55 vs. ≤ 55)	0.97 (0.46–2.04)	0.9331	1.01 (0.48–2.14)	0.9736
Grade (3 vs. 2 vs. 1)	1.73(1.00–3.01)	0.0508	1.29 (0.74–2.24)	0.3646
Size (> 2 vs. ≤ 2 cm)	2.70(1.36–5.36)	4.59E–03	2.28 (1.12–4.66)	0.0233

Taken together, the above results demonstrated that the drug-free prognostic signature could robustly predict recurrent risk of ER+ breast cancer patients after surgery.

### Tamoxifen therapy benefit predictive signature

**Figure 2:**
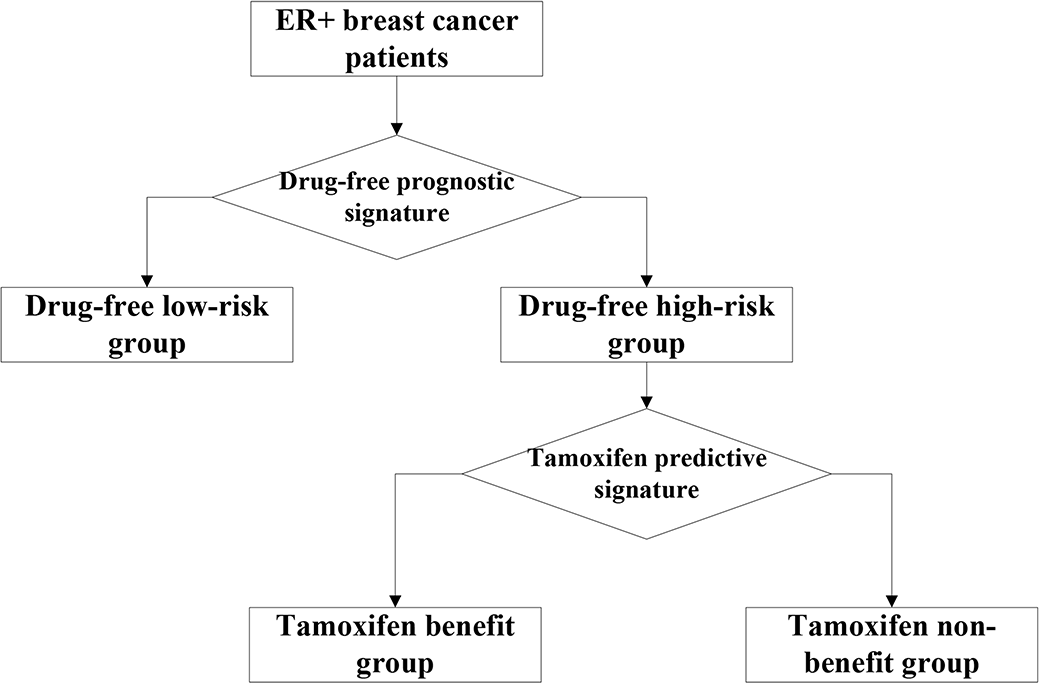
Utilizing the two coupled signatures to identify three groups The two coupled signatures: the drug-free prognostic signature of post-operative recurrent risk and the tamoxifen therapy benefit predictive signature. Three groups: drug-free low-risk group, tamoxifen benefit group and tamoxifen non-benefit group.

For samples of ER+ breast cancer patients receiving post-operative tamoxifen therapy, we firstly used the drug-free prognostic signature to recognize patients who would have been at low risk of recurrence if they had accepted surgery only, and then used the remained high-risk samples to develop a therapy benefit predictive signature for identifying patients who could benefit from tamoxifen therapy (Figure 2).

Notably, the datasets of patients receiving post-operative tamoxifen therapy also included samples of lymph-node-positive patients (Table 2). Under the assumption that both lymph-node-positive and lymph-node-negative patients with high risk of recurrence after surgery would be the same likely to have micro-distant-metastases, we pooled high-risk patients predicted from both lymph-node-positive and lymph-node-negative patients together as the discovery cohort. For each of the four datasets including both lymph-node negative and positive samples, we found no differentially expressed genes (DEGs) between the high-risk patients of the lymph-node positive and negative group using Student's *t*-test, with FDR < 5%. Similarly, no DEGs were found between the low-risk patients of the lymph-node positive and negative group. On the other hand, we found that DEGs between the high- and low-risk groups for lymph-node negative patients was consistent with the corresponding DEGs for lymph-node positive patients. From the GSE17705 dataset, we detected 7075 and 6221 DEGs between the low- and high-risk groups for the lymph-node negative and positive patients, respectively. The two lists of DEGs shared 5312 genes and they all showed the same deregulation directions (up- or down-regulation) in the high-risk patients compared with the low-risk patients, which was highly unlikely to occur by chance (*p* < 1.0E-16, binomial distribution test). Similarly, for the GSE6532_tt1, GSE6532_tt2 and GSE4922_tt datasets, the DEGs between the distinct prognostic groups for lymph-node negative patients were also highly consistent with the corresponding DEGs for lymph-node positive patients (all *p* < 1.0E–16, binomial distribution test). These results provided evidence that the drug-free prognostic signature was independent of the lymph-node status.

Applying the drug-free prognostic signature to a total 521 samples of ER+ breast cancer patients receiving post-operative tamoxifen therapy, collected in the GSE17705, GSE12093 and GSE6532_tt1 datasets, we recognized a total 320 high-risk patients (184, 68 and 68 in the three datasets, respectively). These 320 patients who would have been at high-risk of recurrence if they had accepted surgery only were used as discovery cohort to develop a tamoxifen therapy predictive signature. Then, we developed the tamoxifen therapy benefit predictive signature in the same way of developing the drug-free prognostic signature. Briefly, we firstly identified 89 RFS-relevant pathways using the univariate Cox proportional-hazard model with FDR < 5% (Supplementary Table 2), and from which we further extracted 46 strongly correlated pathways that could be connected together as a large network by linking every two significantly correlated pathways whose Spearman rank correlation coefficient was larger than 0.6 (Supplementary Figure 2). We defined the 46 pathways as the core tamoxifen-associated RFS-relevant pathways and some references suggesting their relevance to tamoxifen resistance were listed in Supplementary Table 2.

**Figure 3:**
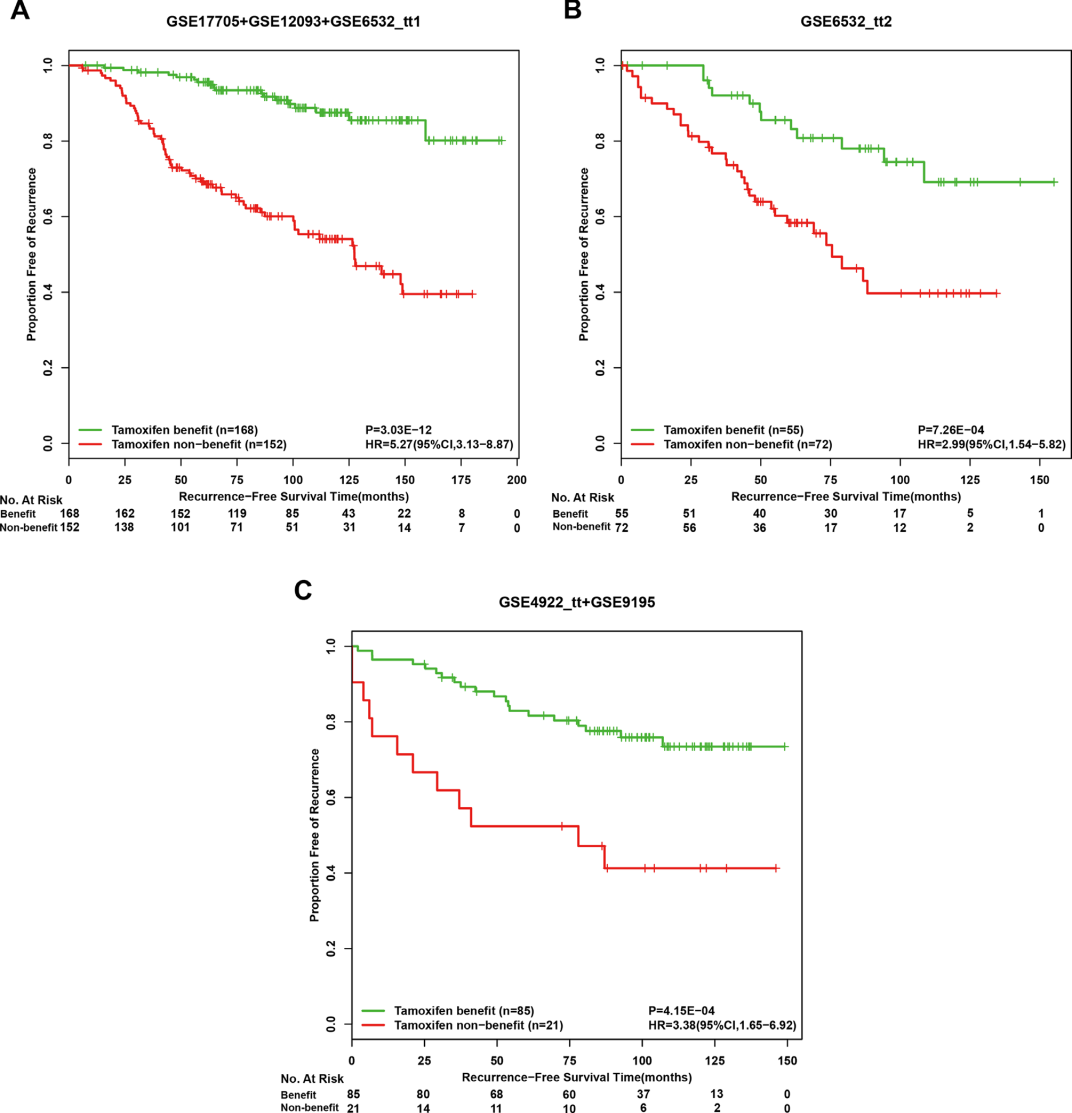
Kaplan-Meier estimates of recurrence-free survival in post-operative tamoxifen-treated patients of drug-free high-risk groups according to the tamoxifen therapy benefit predictive signature Recurrence-free survival curves in the discovery cohort (**A**), the first validation cohort (**B**) and the second validation cohort (**C**).

From 10,096 gene pairs with stable REOs within these 46 pathways in the normal tissue, we identified 67 gene pairs whose reversal REOs were significantly correlated with poor RFS using the univariate Cox proportional-hazard model with FDR < 10%. From these 67 gene pairs, we performed a forward-stepwise selection algorithm to extract a subset of gene pairs with the highest C-index based on the following classification rule: patients were assigned to the tamoxifen benefit group if no gene pair in the subset was reversed and all the other were assigned to the tamoxifen non-benefit group. Finally, a tamoxifen therapy benefit predictive signature consisting of ten gene pairs (Table 5) was identified, which allocated the 320 drug-free high-risk patients into a tamoxifen benefit group of 168 patients and a tamoxifen non-benefit group of 152 patients, respectively. The RFS of the former was significantly better than that of the latter (HR = 5.27, 95%CI:3.13–8.87, *p* = 3.03E-12, C-index = 0.70 Figure 3A).

**Table 5:** The tamoxifen therapy benefit predictive signature

	Gene A			Gene B	
Gene ID	Gene Symbol	Gene Full Name	Gene ID	Gene Symbol	Gene Full Name
1843	DUSP1	dual specificity phosphatase 1	983	CDK1	cyclin-dependent kinase 1
8440	NCK2	NCK adaptor protein 2	983	CDK1	cyclin-dependent kinase 1
2908	NR3C1	nuclear receptor subfamily 3, group C, member 1 (glucocorticoid receptor)	58	ACTA1	actin, alpha 1, skeletal muscle
2625	GATA3	GATA binding protein 3	581	BAX	BCL2-associated X protein
1845	DUSP3	dual specificity phosphatase 3	7204	TRIO	trio Rho guanine nucleotide exchange factor
8878	SQSTM1	sequestosome 1	835	CASP2	Caspase 2, apoptosis-related cysteine peptidase
8660	IRS2	insulin receptor substrate 2	5153	PDE1B	phosphodiesterase 1B, calmodulin-dependent
6196	RPS6KA2	ribosomal protein S6 kinase, 90kDa, polypeptide 2	30849	PIK3R4	phosphoinositide-3-kinase, regulatory subunit 4
1997	ELF1	E74-like factor 1 (ets domain transcription factor)	983	CDK1	cyclin-dependent kinase 1
9146	HGS	Hepatocyte growth factor-regulated tyrosine kinase substrate	983	CDK1	cyclin-dependent kinase 1

Gene A has a higher expression level than Gene B in normal breast tissues.

**Figure 4:**
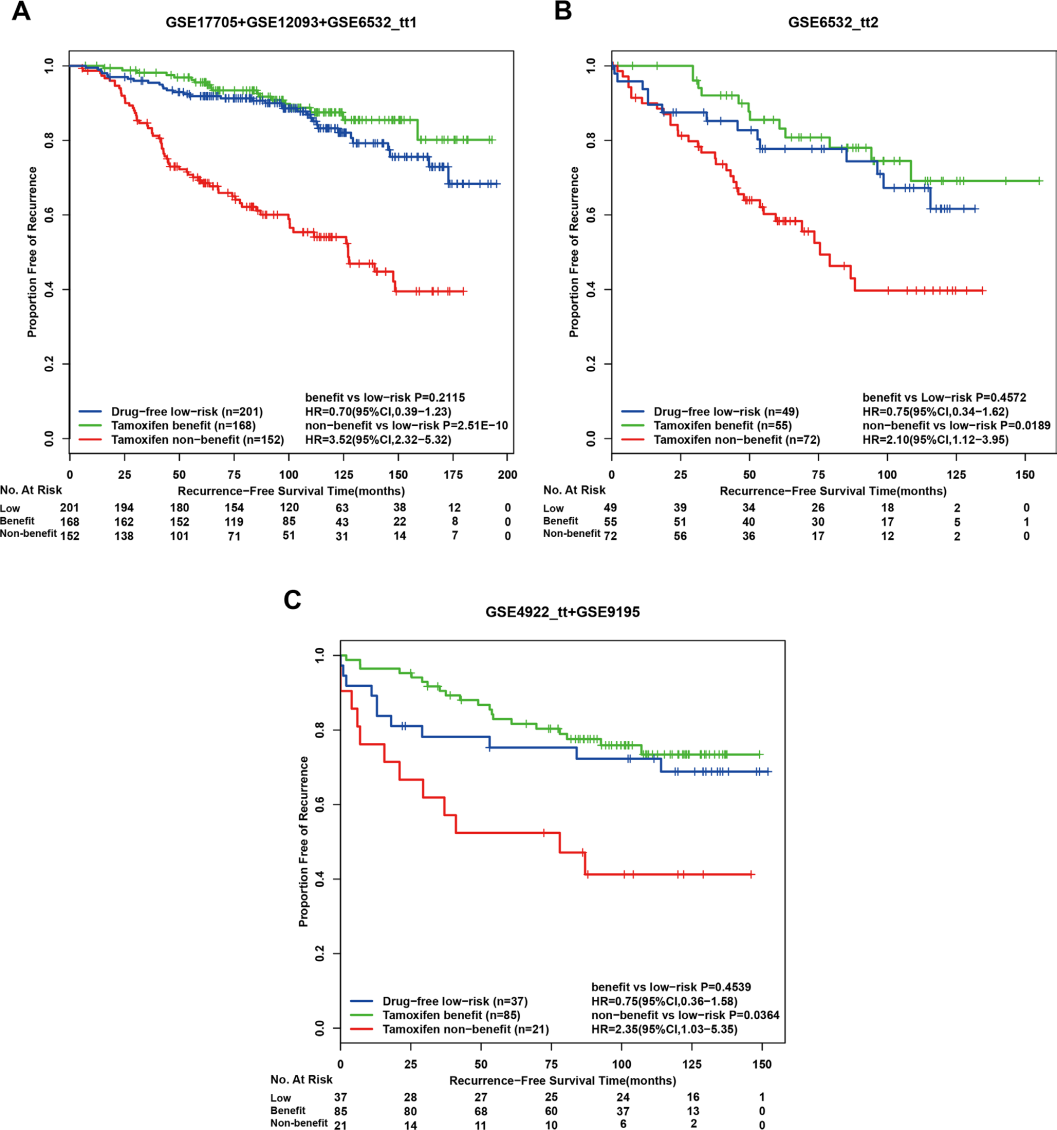
Kaplan-Meier estimates of recurrence-free survival in post-operative tamoxifen-treated patients according to the two coupled signatures Recurrence-free survival curves in the discovery cohort (**A**), the first validation cohort (**B**) and the second validation cohort (**C**). benefit: tamoxifen benefit group; low-risk, drug-free low-risk group; non-benefit: tamoxifen non-benefit group.

In the first independent validation dataset GSE6532_ tt2, for the 127 high-risk patients recognized by the drug-free prognostic signature, 55 and 72 patients were classified into tamoxifen benefit and non-benefit groups, respectively, and the former had a significantly different RFS from the latter (HR = 2.99, 95%CI:1.54–5.82, *p* = 7.26E-04, C-index = 0.64 Figure 3B). From the independent GSE4922_tt and GSE9195 datasets, 34 and 72 drug-free high-risk patients were recognized by the drug-free prognostic signature, respectively, and we pooled them together as the second validation cohort. The therapy benefit predictive signature could stratify this validation cohort into a tamoxifen benefit group of 85 patients and a tamoxifen non-benefit group of 21 patients with significantly different RFS (HR = 3.38, 95%CI:1.65–6.92, *p* = 4.15E–04, C-index = 0.63 Figure 3C). In addition, for each of the discovery and validation cohorts, the RFS of the tamoxifen benefit group was not significantly different from that of the drug-free low-risk group recognized by the drug-free prognostic signature, while the latter group also had significantly better RFS than the tamoxifen non-benefit group (Figure 4). The similar results were observed when applying the two coupled signatures to lymph-node-negative and lymph-node-positive patients separately (Supplementary Table 3). A multivariate analysis in the discovery cohort was not performed due to a number of missing values, while multivariate Cox analyses for the two validation cohorts both showed that the therapy benefit predictive signature remained significantly associated with RFS after adjusting for clinical factors of age, node status, tumor size and histology grade (Table 6).

**Table 6:** Univariate and multivariate Cox regression analysis for the Tamoxifen therapy benefit predictive signature

	Univariate model		Multivariate model	
Variables	HR (95%CI)	*P*	HR(95%CI)	*P*
The 106 samples of the first validation cohort				
The ten gene pairs	3.35 (1.56–7.19)	1.97e–03	2.49 (1.12–5.53)	0.0246
Age (> 55 vs. ≤ 55)	0.63 (0.30–1.31)	0.2191	0.52 (0.23–1.13)	0.0998
Grade (3 vs. 2 vs. 1)	1.45 (0.84–2.53)	0.1856	1.24 (0.68–2.26)	0.4907
Size (> 2 vs. ≤ 2 cm)	2.84 (1.24–6.51)	0.0138	2.57 (1.06–6.22)	0.0368
Node (positive vs. negative)	1.32 (0.68–2.59)	0.4131	1.16 (0.57–2.35)	0.6786
The 88 samples of the second validation cohort				
The ten gene pairs	3.08 (1.49–6.35)	2.38e–03	3.42 (1.64–7.13)	0.0010
Age (> 55 vs. ≤ 55)	1.48 (0.57–3.87)	0.4194	1.66 (0.58–4.72)	0.3431
Grade (3 vs. 2 vs. 1)	1.47 (0.87–2.48)	0.1476	1.19 (0.63–2.26)	0.5874
Size (> 2 vs. ≤ 2 cm)	2.53 (1.04–6.17)	0.0413	1.76 (0.66–4.67)	0.2565
Node (positive vs. negative)	2.60 (1.16–5.83)	0.0200	2.62 (1.16–5.92)	0.0201

**Figure 5:**
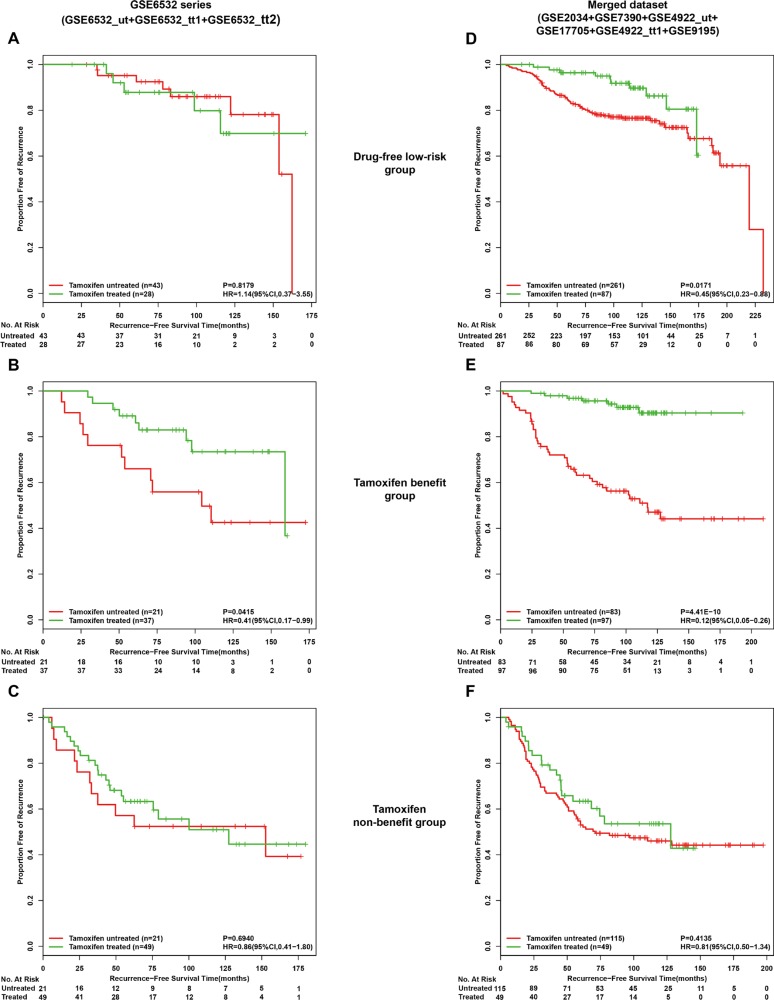
Kaplan-Meier analysis of recurrence-free survival as a function of tamoxifen treatment in different risk groups of lymph-node-negative patients From the GSE6532 series (GSE6532_ut,GSE6532_tt1 and GSE6532_tt2), recurrence-free survival curves in drug-free low risk group (**A**), tamoxifen benefit group (**B**) and tamoxifen non-benefit group (**C**). From a merged dataset including 233 lymph-node-negative patients receiving post-operative tamoxifen therapy (GSE17705, GSE4922_tt and GSE9195) and 459 lymph-node-negative patients accepting surgery only (GSE2034, GSE7390 and GSE4922_ut), recurrence-free survival curves in (**D**), (**E**) and (**F**) corresponding to (**A**), (**B**) and (**C**).

The GSE6532 series included 85 samples of lymph-node-negative patients accepting surgery only (GSE6532_ut) and 114 lymph-node-negative patients treated with tamoxifen (GSE6532_tt1 and GSE6532_tt2). Thus, we could compared RFS between the tamoxifen-treated and the tamoxifen-untreated patients in each of the three groups classified by the two coupled signatures. As expected, in the drug-free low-risk group, RFS of the 28 tamoxifen-treated patients were not significantly different from that of the 43 tamoxifen-untreated patients (HR = 1.14, 95%CI:0.37–3.55, *p* = 0.8179, Figure 5A). Also, in the tamoxifen non-benefit group, the 49 tamoxifen-treated patients had no significant better RFS than the 21 tamoxifen-untreated patients (HR = 0.86, 95%CI:0.41 amoxifen- untreated p1.79, *p* = 0.6940, Figure 5C). These results suggested that both the drug-free low-risk patients and the tamoxifen non-benefit patients could not benefit from tamoxifen therapy. In contrast, in the tamoxifen benefit group, the 37 tamoxifen-treated patients had a significant better RFS than the 21 tamoxifen-untreated patients (HR = 0.41, 95%CI:0.17–0.99, *p* = 0.0415, Figure 5B). Similar comparison results were found in a merged dataset that included 233 samples of lymph-node-negative patients receiving post-operative tamoxifen therapy (GSE17705, GSE4922_tt and GSE9195) and 459 samples of lymph-node-negative patients accepting surgery only (GSE2034, GSE7390 and GSE4922_ut) (Figure 5D, 5E, 5F). This comparison analysis could not be performed for lymph-node-positive patients because there were no samples of lymph-node-positive patients without accepting tamoxifen therapy.

Taken together, the above results suggested that the two coupled signatures could be used to facilitate the clinical decision of tamoxifen therapy.

## DISCUSSION

In this study, we identified a therapy benefit predictive signature coupled with a drug-free prognostic signature for early stage ER+ breast cancer patients. The two signatures can be used sequentially to stratify early stage ER+ breast cancer patients into three groups. The first group includes patients who will be at low-risk of recurrence if they accept surgery only, and we could recommend them to accept no or a short duration of tamoxifen treatment. The second group includes patients who will be at high risk of post-operative recurrence but can benefit from tamoxifen therapy. For these patients, the decreased risk after tamoxifen therapy could be attributed to the tamoxifen efficacy, and thus tamoxifen therapy could be recommended to them. For the third group of patients who will keep at high risk after tamoxifen therapy, we can infer that the routine clinical tamoxifen therapy cannot improve their clinical outcomes. Different from previously reported prognostic signature, the two coupled signatures can find most of patients who could benefit from tamoxifen therapy and the patients at low risk with surgery only, and thus insulating them from cytotoxic chemotherapy or even tamoxifen therapy.

Notably, for the third group of patients, we should not simply infer that they are resistant to (or cannot respond to) tamoxifen. Some of these patients, who could have poor prognoses on account of their resistance to drug-induced tumor cell apoptosis [48], could be considered to be truly resistant to tamoxifen, so prescription of other treatment modalities such as chemotherapies or target therapies could be recommended [49, 50]. However, a large portion of these patients could respond to tamoxifen but the therapy efficacy may be insufficient in competition with tumor growth ability [51, 52]. If this is the case, a larger dosage and longer duration of tamoxifen therapy could be recommended [53]. Thus, the therapy benefit predictive signature can be regarded as an apparently resistant signature which can be used to predict whether the prognosis of a patient can be improved by the routine clinical tamoxifen therapy. To identify a drug resistant signature for discriminating patients who can respond to tamoxifen, we need gene expression data of responders an non-responders of patients accepting tamoxifen therapy, which, however, are currently unavailable for post-operative patients. Nevertheless, samples of metastatic patients accepting tamoxifen therapy, whose response to the treatment can be clearly defined [54], could be subjected to gene expression profiling to develop the drug resistant signature.

In clinical practice, almost all lymph-node positive patients undergo lymphadenectomy [55] and after that they should have low risk of recurrence if they have no micro-distant-metastases. We assumed that high-risk patients predicted from either the lymph-node negative or positive group by the drug-free prognostic signature would be the same likely to have micro-distant-metastases. Thus, the signature should be independent of the lymph-node status, as evidenced by the observation that the transcriptome difference between the distinct prognostic groups for lymph-node negative samples was consistent with the corresponding difference for lymph-node positive samples and no transcriptome difference could be observed between the same prognostic groups predicted from the lymph-node positive and negative patients. All of these suggested that high-risk patients of the lymph-node positive and negative group possess similar molecular characteristics.

For clinical application, we can develop a custom array or RT-PCR kit to measure expression intensities of the 32 genes included in the two coupled signatures to determine the REOs of the signature gene pairs. Compared with the microarray technique, the RT-PCR technique is more reliable and reproducible for quantitation of transcriptional abundance of genes. Notably, the problem of experimental batch effect and data normalization also exists when RT-PCR is used to measure gene expression intensities [56]. However, it can be expected that REOs deduced from gene intensities measured by RT-PCR tend to be robust against experimental batch effects.

Due to the high-dimension problem inherent in microarray data, especially when we focus on analyzing a huge number of gene pairs, the identification of disease signatures is liable to false discoveries [27]. Through mapping gene pairs into pathways, we started with pathways to improve the robustness of the identification of signatures. As demonstrated in this study, the identified signatures can perform robustly in independent datasets. However, due to the limited gene annotation to biological pathways [57, 58], some important pathways associated with survival might be missed. A method worth exploring is to augment annotated genes of pathways using genes that are closely linked with intra-pathway genes in protein-protein interaction network [59, 60].

In this study, in order to ensure the robustness of signature performance in samples detected by different Affymetrix platforms, GPL96 and GPL570, we defined stable gene pairs commonly detected by the two platforms as the ultimate stable gene pairs. Because different platforms have different probe designs and experimental protocol, some gene pairs may not keep consistent REOs in different platforms. Further study is needed to evaluate whether the two coupled signatures indentified in this study are suitable for microarray data produced by other platforms.

## MATERIALS AND METHODS

### Data and pre-processing

All gene expression datasets for normal breast tissue and ER+ breast cancer were collected from GEO [61], as described in detail in Table 1. All samples used in this study fell into three categories: samples of normal breast tissue for identifying gene pairs with stable REOs in normal breast tissue, samples of ER+ lymph-node-negative breast cancer patients accepting surgery only for developing a drug-free prognostic signature and samples of post-operative tamoxifen-treated ER+ breast cancer patients for developing a therapy benefit predictive signature. The third category included both lymph-node-negative and lymph-node-positive patients, while most of them are in early stage (Table 2). RFS served as the prognosis endpoint, representing both disease-free survival and distant metastasis-free survival [47].

All the above-mentioned data were produced by the GPL96 or GPL570 platform. For each of the datasets, raw intensity files (.CEL) were processed using the RMA algorithm for background adjustment and median polish summarization without quantile normalization [62]. With the custom CDF file, each probe set ID was mapped to Gene ID, and then probe sets that mapped to multiple Gene IDs or did not map to any Gene ID were removed. The expression measurements of all probe sets corresponding to the same Gene ID were averaged to obtain a single measurement (on the log2 scale). The raw mRNA expression data of the post-operative tamoxifen treated patients were processed with the RMA quantile normalization algorithm in order to select DEGs between the high- and low-risk patients predicted by the drug-free prognostic signature.

The annotation data of 1320 canonical pathways, covering 8428 unique genes, were downloaded from the C2 collection of MSigDB (Version 4.0, updated May 31, 2013) [63] for personalized pathway analysis.

### Consistency evaluation of stable REOs detected by different platforms

We focused on analyzing the 12752 genes measured by both the GPL96 and GPL570 platforms. For a collection of normal breast samples measured by a particular platform, if gene *A* had a higher (or smaller) expression level than gene *B* in more than 99% normal samples, then the gene pair *(A,B)* was defined as stable gene pair. Based on the overlapping stable gene pairs detected by both the GPL96 and GPL570 platforms, a consistency score was calculated as the percentage of stable gene pairs with identical REOs in both collections of normal samples. We evaluated whether the consistency score was higher than what expected by chance using the binomial distribution test as following:

$$p=1-\sum_{i=0}^{k-1}\left(\begin{array}{c} n\\ i \end{array}\right)0.5^{i}\;{\left(1-0.5\right)}^{n-i}$$ (1)

where 0.5 is the probability of observing a gene pair having the same REO in two collections of normal samples by chance, *n* denotes the number of overlapping stable gene pairs detected by the two platforms, and *k* denotes the number of stable gene pairs with identical REOs in the two collections of normal samples.

### Survival analysis

The univariate Cox proportional-hazards model [64] was used to evaluate the correlation of disruption indexes of pathways with the RFS and to evaluate whether a gene pair's reversal REOs were significantly correlated with poor RFS. When identifying RFS relevant gene pairs, we characterized REO of intra-pathway gene pairs for each sample as a binary vector in which 0 represented the REO of the intra-pathway gene pair in a cancer sample in line with that in normal tissue while 1 represented reversal REO. Kaplan-Meier survival plots and log-rank tests [65] were used to evaluate the differences in RFS of distinct groups. The Cox proportional-hazards model was also performed to calculate the hazard ratios (HRs) and their 95% confidence intervals (CIs). The independent prognostic value of a signature was assessed by multivariate Cox proportional-hazards model. To evaluate the predictive performance of a signature we adopted the concordance index (C-index), which is a measure of overall concordance between predicted risk scores and observed RFS [66]. C-index, ranging from 0.5 (indicating random chance) to 1 (indicating perfect discrimination), is one of the most appropriate index for studies focusing on long-term risk prediction [67]. The Benjamini-Hochberg multiple testing correction was used to estimate the false discovery rate (FDR) [68]. All statistical analyses were performed using the R software package version 3.0.1.

### Algorithm for searching optimum signatures

For a set of gene pairs whose REOs were associated with poor RFS, a forward-stepwise selection algorithm was performed to search for a optimal subset of these gene pairs that resulted in the highest C-index. Starting with the intra-pathway gene pair with the largest C-index as the seed signature, candidate intra-pathway gene pairs were added to the signature one at a time until the addition of one gene pair did not improve predictive performance.

## SUPPLEMENTARY MATERIAL TABLES AND FIGURES



## Abbreviations

ER+: Estrogen Receptor-Positive
RFS: Recurrence-Free Survival
REO: Relative Expression Ordering
FDR: False Discovery Rate
HR: Hazard Ratios
CI: Confidence Intervals
C-index: Concordance index.
