# Supplementary material for: Tamoxifen therapy benefit predictive signature coupled with prognostic signature of post-operative recurrent risk for early stage ER+ breast cancer
Source: Oncotarget. 2015 Oct 30;6(42):44593–608. doi: 10.18632/oncotarget.6260 (PMC4792578; doi:10.18632/oncotarget.6260)
Supplement: Supplementary file 1 [file oncotarget-06-44593-s001.pdf]

Tamoxifen therapy benefit predictive signature coupled with prognostic signature of post-operative recurrent risk for early stage ER+ breast cancer

Supplementary Materials

Supplementary Table 1: The 37 drug-free RFS-relevant pathways

Pathway name	Number of Stable gene pairs	*p*	β#	Reference
KEGG: Glycosylphosphatidylinositol (GPI)-anchor biosynthesis	22	< 1.0E–16	2491.39	
KEGG: Pantothenate and CoA biosynthesis	13	9.63E–06	41.16	
**KEGG: Ribosome***	1289	1.24E–03	104.27	[1]
**KEGG: P53 signaling pathway**	333	3.95E–04	9.68	[2]
**KEGG: Ubiquitin mediated proteolysis**	1153	1.99E–04	26.59	[3]
**KEGG: Progesterone mediated oocyte maturation**	449	2.61E–04	17.19	
**BioCarta: AKAP95 pathway**	17	1.26E–03	3.53	
**BioCarta: DNA fragment pathway**	15	1.35E–03	5.15	[4]
BioCarta: TCRA pathway	11	1.15E–04	25.15	
**BioCarta: Stathmin pathway**	15	1.03E–03	3.62	[5]
REACTOME: CTNNB1 phosphorylation cascade	7	3.07E–04	7.85	
**REACTOME: TRIF mediated TLR3 signaling**	451	5.07E–04	16.02	
**REACTOME: G0 and early G1**	61	1.27E–03	5.17	[6]
**REACTOME: Prolonged ERK activation events**	32	1.40E–03	4.51	[7]
**REACTOME: Signalling to RAS**	60	1.38E–03	6.15	[8]
REACTOME: p38 MAPK events	3	5.98E–04	8.02	
REACTOME: Hyaluronan metabolism	3	5.98E–04	8.02	
REACTOME: Phosphorylation of CD3 and TCR zeta chains	17	1.15E–04	38.87	
REACTOME: Translocation of ZAP–70 to immunological synapse	15	1.15E–04	34.30	
REACTOME: p75NTR recruits signalling complexes	15	5.48E–07	51.33	
REACTOME: p75NTR signals via NFkB	24	5.48E–07	82.14	
REACTOME: NFkB is activated and signals survival	17	5.48E–07	58.18	
**REACTOME: Recruitment of mitotic centrosome proteins and complexes**	419	4.08E–04	16.91	[9]
**REACTOME: Loss of Nlp from mitotic centrosomes**	334	9.89E–04	13.81	[10]
REACTOME: Activation of genes by ATF4	56	1.21E–04	62.80	
**REACTOME: Metabolism of mRNA**	8220	7.04E–04	101.45	
REACTOME: Metabolism of RNA	11185	7.42E–04	108.28	

**REACTOME: Mitotic G1-G1/S phases**	2091	8.86E–04	30.77	
**REACTOME: MAP kinase activation in TLR cascade**	109	3.29E–04	5.37	[11]
**REACTOME: NFkB and MAP kinases activation mediated by TLR4 signaling repertoire**	406	4.87E–04	17.01	[12]
REACTOME: Intrinsic pathway	23	9.63E–06	72.82	
REACTOME: Synthesis of very long-chain fatty acyl-CoAs	9	3.27E–06	14.92	
**REACTOME: E2F mediated regulation of DNA replication**	103	2.57E–04	11.02	[13, 14]
**REACTOME: APC/C:Cdc20 mediated degradation of Cyclin B**	31	1.13E–04	7.16	[15]
**REACTOME: Phosphorylation of the APC/C**	16	7.32E–05	3.67	[16]
**REACTOME: Class I MHC mediated antigen processing & presentation**	4306	1.34E–03	44.45	[17]
**REACTOME: Antigen processing: ubiquitination & proteasome degradation**	2485	6.82E–04	35.10	

*Pathways whose names are in bold were regard as the core drug-free RFS-relevant pathways**.**

#βwas obtained from univariate Cox regression analysis.

Supplementary Table 2: The 89 tamoxifen-associated RFS-relevant pathways

Pathway name	Number of stable gene pairs	*p*	β#	References
KEGG: Tyrosine metabolism	132	1.75E–03	12.18	
KEGG: Alpha-linolenic acid metabolism	11	3.33E–04	13.80	
KEGG: Pantothenate and CoA biosynthesis	13	5.79E–05	19.18	
**KEGG: Oocyte meiosis***	912	3.09E–03	12.50	
**KEGG: Progesterone mediated oocyte maturation**	449	6.01E–05	20.45	
KEGG: Dilated cardiomyopathy	739	1.43E–03	15.64	
BioCarta: NO1 pathway	134	8.48E–04	8.97	
**BioCarta: Srcrptp pathway**	16	1.03E–04	5.69	[18]
**BioCarta: AKAP95 pathway**	17	6.93E–04	4.67	
**BioCarta: G2 pathway**	64	1.81E–03	8.06	[19]
**BioCarta: Cellcycle pathway**	44	7.84E–05	11.48	[20]
BioCarta: Salmonella pathway	25	8.05E–04	4.07	
**BioCarta: MPR pathway**	164	4.49E–07	15.94	
BioCarta: Integrin pathway	118	8.46E–05	7.25	
BioCarta: mCalpain pathway	62	1.15E–04	5.39	
BioCarta: NFAT pathway	292	1.78E–04	6.67	
**BioCarta: Akapcentrosome pathway**	19	1.60E–04	6.57	[21]
BioCarta: Rab pathway	18	3.36E–03	2.84	
BioCarta: MAL pathway	48	1.75E–04	7.21	
BioCarta: SODD pathway	2	3.74E–07	5.50	
**BioCarta: Ptc1 pathway**	15	2.15E–05	5.48	
BioCarta: Barrestin pathway	16	1.16E–03	14.90	
**BioCarta: Stathmin pathway**	15	1.40E–04	4.82	[22]
BioCarta: HSP27 pathway	23	5.56E–05	5.48	

BioCarta: uCalpain pathway	48	3.70E–04	10.79	
BioCarta: Actiny pathway	58	1.57E–04	6.44	
ST: Tumor necrosis factor pathway	74	2.65E–03	13.91	
ST: Type I interferon pathway	5	1.01E–08	6.10	
PID: RhoA pathway	270	4.79E–04	9.88	
PID: Ret pathway	112	3.09E–03	14.55	
**PID: Reg GR pathway**	691	1.82E–03	24.22	[23]
**PID: AP1 pathway**	582	1.37E–05	22.96	[24]
**PID: Retinoic acid pathway**	74	4.81E–04	11.62	[25]
PID: Caspase pathway	236	8.92E–04	12.29	
PID: Integrin A4B1 pathway	99	9.00E–04	7.66	
PID: FAK pathway	301	2.96E–03	11.01	
REACTOME: Glycogen breakdown glycogenolysis	19	3.30E–03	9.12	
**REACTOME: Signalling by NGF**	3973	1.00E–03	34.20	[26]
**REACTOME: TRIF mediated TLR3 signaling**	451	1.57E–04	22.45	
REACTOME: IL 7 Signaling	14	1.22E–03	17.86	
**REACTOME: Signaling by ERBB4**	703	1.95E–03	19.37	[27]
**REACTOME: Signaling by ERBB2**	894	8.51E–04	29.70	[28]
**REACTOME: GRB2 events in ERBB2 signaling**	32	6.40E–04	6.14	
**REACTOME: Signaling by EGFR in cancer**	990	6.47E–05	30.47	[29]
**REACTOME: SHC1 events in ERBB4 signaling**	33	6.49E–05	7.30	[30]
REACTOME: RORA activates circadian expression	30	8.67E–04	9.32	
**REACTOME: ARMS mediated activation**	28	3.71E–04	5.84	[31]
**REACTOME: Prolonged ERK activation events**	32	5.59E–04	5.60	[32]
**REACTOME: Signalling to Ras**	60	1.52E–04	8.03	[33]
**REACTOME: NGF signalling via TRKA from the plasma membrane**	1360	9.78E–05	25.86	[26]
**REACTOME: Signalling to ERKS**	93	3.04E–04	9.21	[34]
**REACTOME: Signaling by FGFR in disease**	1331	1.45E–04	38.12	[35]
**REACTOME: Signalling to p38 via RIT and RIN**	23	8.68E–04	5.28	
REACTOME: Acyl chain remodelling of PC	21	1.79E–03	10.83	
REACTOME: Synthesis of PA	40	1.90E–03	40.25	
REACTOME: Acyl chain remodelling of PG	4	3.33E–04	5.02	
REACTOME: Acyl chain remodelling of PE	21	1.79E–03	10.83	
REACTOME: Acyl chain remodelling of PS	12	1.79E–03	6.19	
REACTOME: MicroRNA miRNA biogenesis	31	9.41E–04	21.32	
REACTOME: Nuclear events kinase and transcription factor activation	8	1.33E–03	3.95	
REACTOME: Regulatory RNA pathways	32	9.41E–04	22.01	
**REACTOME: SHC1 events in EGFR signaling**	13	1.24E–04	2.77	[33]
REACTOME: ERK/MAPK targets	7	1.52E–03	3.99	
REACTOME: Processive synthesis on the lagging strand	14	1.22E–03	35.71	
REACTOME: Striated muscle contraction	79	1.93E–03	7.91	
**REACTOME: Downstream signal transduction**	660	1.70E–04	21.91	
REACTOME: Muscle contraction	301	9.78E–04	6.87	

**REACTOME: Cyclin A/B1 associated events during G2/M transition**	23	9.27E–04	3.66	
**REACTOME: Downstream signaling of activated FGFR**	862	1.37E–03	27.80	[36]
**REACTOME: MAP kinase activation in TLR cascade**	109	1.09E–05	9.07	
REACTOME: cGMP effects	16	1.71E–04	7.45	
REACTOME: Nitric oxide stimulates guanylate cyclase	21	5.42E–06	7.72	
**REACTOME: TRAF6 mediated induction of NFkB and MAP kinases upon TLR7/8 OR 9 activation**	459	7.14E–05	26.73	
**REACTOME: NFkB and MAP kinases activation mediated by TLR4 signaling repertoire**	406	2.85E–05	26.11	
**REACTOME: Signaling by insulin receptor**	1110	2.09E–03	32.49	[37]
REACTOME: Purine metabolism	46	1.18E–03	16.17	
**REACTOME: SOS mediated signalling**	19	6.03E–04	2.65	
**REACTOME: RAF/MAP kinase cascade**	8	2.50E–04	2.33	[38]
**REACTOME: SHC mediated signalling**	17	1.54E–04	3.58	[39]
**REACTOME: MyD88:MAL Cascade initiated on plasma membrane**	527	1.20E–04	28.15	
**REACTOME: APC/C:Cdc20 mediated degradation of Cyclin B**	31	6.62E–04	3.82	
REACTOME: Chylomicron mediated lipid transport	17	2.56E–04	47.15	
**REACTOME: Activated TLR4 signalling**	611	6.15E–04	24.24	
**REACTOME: Phosphorylation of the APC/C**	16	1.03E–04	3.19	
REACTOME: Complement cascade	80	9.50E–04	28.65	
**REACTOME: Toll receptor cascades**	924	2.83E–03	26.20	
**REACTOME: G2/M Checkpoints**	143	1.93E–03	6.05	
**REACTOME: Signaling by FGFR**	1097	1.60E–04	37.96	[35, 36]
**REACTOME: SHC-related events**	32	1.64E–04	5.94	[39]

*Pathways whose names are in bold were regard as the core tamoxifen-associated RFS-relevant pathways. #βwas obtained from univariate Cox regression analysis.

Supplementary Table 3: Comparison of recurrence-free survival between three groups identified by the two coupled signatures in lymph-node-negative and lymph-node-positive patients receiving post-operative tamoxifen therapy separately

	Low	High	HR (95%CI)	*P*	C-index
Lymph-node-negative patients of the discovery cohort					
benefit vs. low-risk	79	70	0.93 (0.33–2.65)	0.8934	0.48
non-benefit vs. low-risk	79	55	4.93 (2.26–10.75)	9.17E–06	0.72
non-benefit vs. benefit	70	55	6.01 (2.43–14.87)	1.02E–05	0.73
Lymph-node-negative patients of the validation cohort*					
benefit vs. low-risk	36	64	0.94 (0.34–2.60)	0.9105	0.52
non-benefit vs. low-risk	36	43	4.52 (1.79–11.42)	5.34E–04	0.69
non-benefit vs. benefit	64	43	4.91 (2.27–10.64)	9.21E–06	0.70
Lymph-node-positive patients of the discovery cohort					
benefit vs. low-risk	52	53	0.84 (0.37–1.91)	0.6824	0.49
non-benefit vs. low-risk	52	65	2.85 (1.52–5.32)	6.13E–04	0.63
non-benefit vs. benefit	53	65	3.53 (1.75–7.14)	1.82E–04	0.64
Lymph-node-positive patients of the validation cohort*					
benefit vs. low-risk	45	72	0.71 (0.38–1.31)	0.2681	0.44
non-benefit vs. low-risk	45	47	1.46 (0.79–2.67)	0.2224	0.53
non-benefit vs. benefit	72	47	2.19 (1.23–3.92)	6.52E–03	0.61

*The validation cohort was composed of GSE6532_tt2,GSE4922_tt and GSE9195 datasets. benefit: tamoxifen benefit group; low-risk, drug-free low-risk group; non-benefit: tamoxifen non-benefit group.

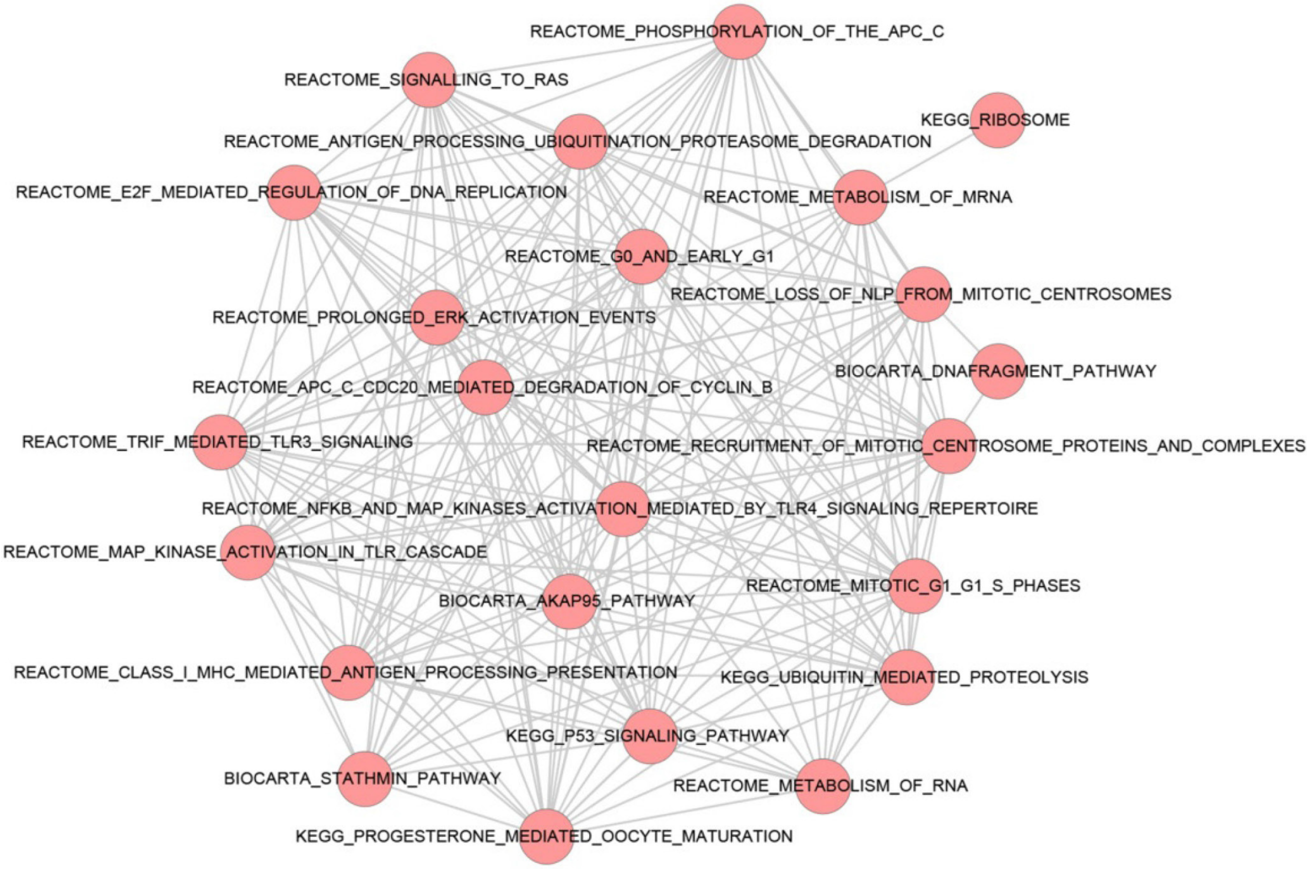

Supplementary Figure 1: The network of the 23 core drug-free RFS-relevant pathways. Node labels represent standard names of the pathways documented in MSigDB. Two significantly correlated pathways, whose Spearman rank correlation coefficient was larger than 0.6, were linked by an edge.

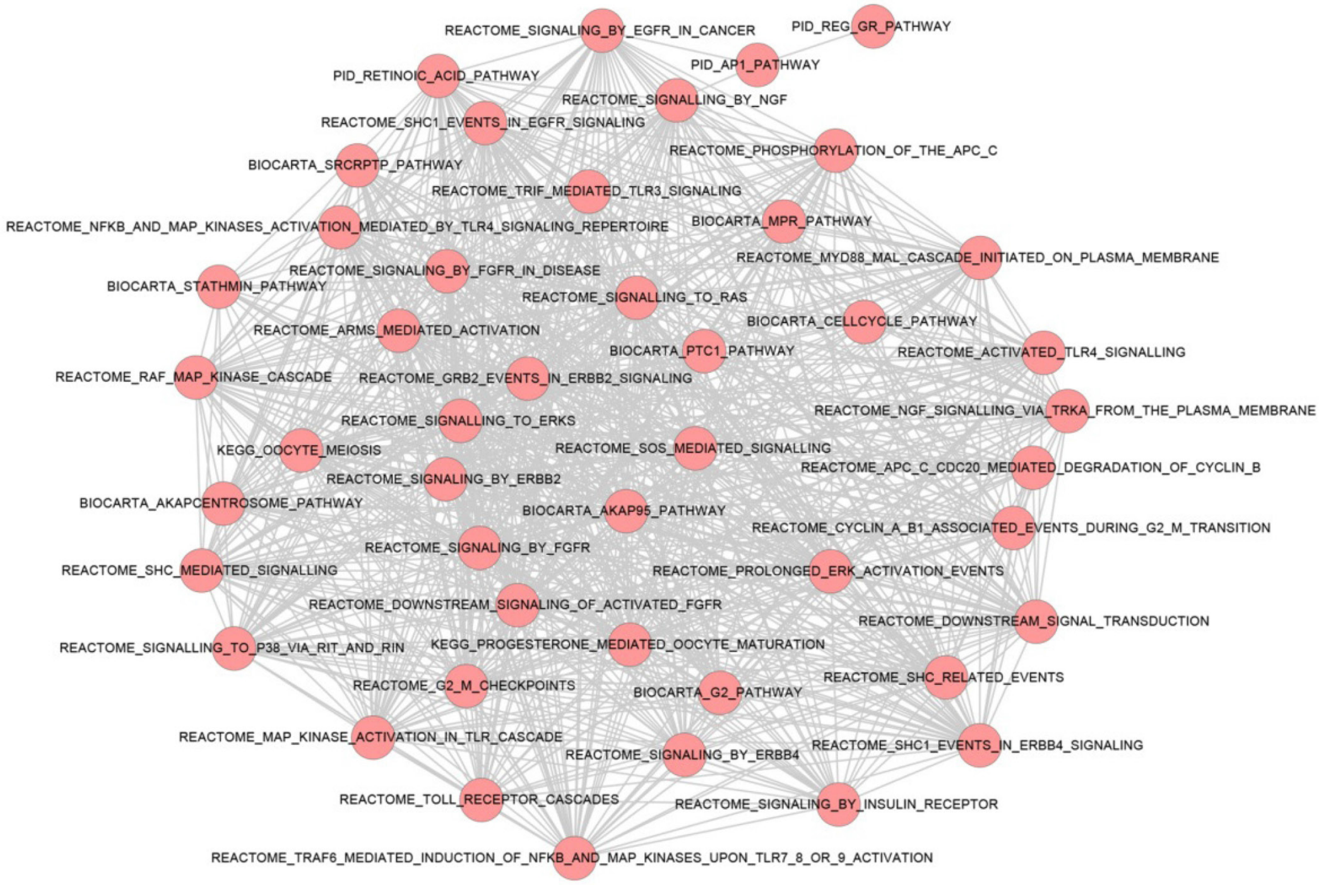

Supplementary Figure 2: The network of the 46 core tamoxifen-associated RFS-relevant pathways. Node labels represent standard names of the pathways documented in MSigDB. Two significantly correlated pathways, whose Spearman rank correlation coefficient was larger than 0.6, were linked by an edge.

REFERENCES

1. de Las Heras-Rubio A, Perucho L, Paciucci R, Vilardell J, ME LL. Ribosomal proteins as novel players in tumorigenesis. Cancer Metastasis Rev. 2014; 33:115–141.
2. Powell E, Piwnica-Worms D, Piwnica-Worms H. Contribution of p53 to metastasis. Cancer Discov. 2014; 4:405–414.
3. Jang KW, Lee KH, Kim SH, Jin T, Choi EY, Jeon HJ, Kim E, Han YS, Chung JH. Ubiquitin ligase CHIP induces TRAF2 proteasomal degradation and NF-kappaB inactivation to regulate breast cancer cell invasion. J Cell Biochem. 2011; 112:3612–3620.
4. Synowiec E, Stefanska J, Morawiec Z, Blasiak J, Wozniak K. Association between DNA damage, DNA repair genes variability and clinical characteristics in breast cancer patients. Mutat Res. 2008; 648:65–72.
5. Baquero MT, Hanna JA, Neumeister V, Cheng H, Molinaro AM, Harris LN, Rimm DL. Stathmin expression and its relationship to microtubule-associated protein tau and outcome in breast cancer. Cancer. 2012; 118:4660–4669.
6. Wang S, Melkoumian Z, Woodfork KA, Cather C, Davidson AG, Wonderlin WF, Strobl JS. Evidence for an early G1 ionic event necessary for cell cycle progression and survival in the MCF-7 human breast carcinoma cell line. J Cell Physiol. 1998; 176:456–464.
7. Zhou YH, Liao SJ, Li D, Luo J, Wei JJ, Yan B, Sun R, Shu Y, Wang Q, Zhang GM, Feng ZH. TLR4 ligand/H(2)O(2) enhances TGF-beta1 signaling to induce metastatic potential of non-invasive breast cancer cells by activating non-Smad pathways. PLoS One. 2013; 8:e65906.
8. Larive RM, Moriggi G, Menacho-Marquez M, Canamero M, de Alava E, Alarcon B, Dosil M, Bustelo XR. Contribution of the R-Ras2 GTP-binding protein to primary breast tumorigenesis and late-stage metastatic disease. Nat Commun. 2014; 5:3881.
9. Ogden A, Rida PC, Aneja R. Heading off with the herd: how cancer cells might maneuver supernumerary centrosomes for directional migration. Cancer Metastasis Rev. 2013; 32:269–287.

10. Li J, Zhan Q. The role of centrosomal Nlp in the control of mitotic progression and tumourigenesis. Br J Cancer. 2011; 104:1523–1528.

11. Bhatelia K, Singh K, Singh R. TLRs: linking inflammation and breast cancer. Cell Signal. 2014; 26:2350–2357.

12. Yang H, Wang B, Wang T, Xu L, He C, Wen H, Yan J, Su H, Zhu X. Toll-like receptor 4 prompts human breast cancer cells invasiveness via lipopolysaccharide stimulation and is overexpressed in patients with lymph node metastasis. PLoS One. 2014; 9:e109980.

13. Chiker S, Pennaneach V, Loew D, Dingli F, Biard D, Cordelieres FP, Gemble S, Vacher S, Bieche I, Hall J, Fernet M. Cdk5 promotes DNA replication stress checkpoint activation through RPA-32 phosphorylation, and impacts on metastasis free survival in breast cancer patients. Cell Cycle. 2015:0.

14. Nguyen-Vu T, Vedin LL, Liu K, Jonsson P, Lin JZ, Candelaria NR, Candelaria LP, Addanki S, Williams C, Gustafsson JA, Steffensen KR, Lin CY. Liver x receptor ligands disrupt breast cancer cell proliferation through an E2F-mediated mechanism. Breast Cancer Res. 2013; 15:R51.

15. Winters ZE, Hunt NC, Bradburn MJ, Royds JA, Turley H, Harris AL, Norbury CJ. Subcellular localisation of cyclin B, Cdc2 and p21(WAF1/CIP1) in breast cancer. association with prognosis. Eur J Cancer. 2001; 37:2405–2412.

16. Barford D. Structure, function and mechanism of the anaphase promoting complex (APC/C). Q Rev Biophys. 2011; 44:153–190.

17. Gobbi G, Mirandola P, Micheloni C, Solenghi E, Sponzilli I, Artico M, Soda G, Zanelli G, Pelusi G, Fiorini T, Cocco L, Vitale M. Expression of HLA class I antigen and proteasome subunits LMP-2 and LMP-10 in primary vs. metastatic breast carcinoma lesions. Int J Oncol. 2004; 25:1625–1629.

18. Leung KC, Brce J, Doyle N, Lee HJ, Leong GM, Sjogren K, Ho KK. Regulation of growth hormone signaling by selective estrogen receptor modulators occurs through suppression of protein tyrosine phosphatases. Endocrinology. 2007; 148:2417–2423.

19. Viedma-Rodriguez R, Ruiz Esparza-Garrido R, Baiza-Gutman LA, Velazquez-Flores MA, Garcia-Carranca A, Salamanca-Gomez F, Arenas-Aranda D. Involvement of multiple cellular pathways in regulating resistance to tamoxifen in BIK-suppressed MCF-7 cells. Tumour Biol. 2015.

20. Babu RL, Naveen Kumar M, Patil RH, Devaraju KS, Ramesh GT, Sharma SC. Effect of estrogen and tamoxifen on the expression pattern of AP-1 factors in MCF-7 cells: role of c-Jun, c-Fos, and Fra-1 in cell cycle regulation. Mol Cell Biochem. 2013; 380:143–151.

21. Lee HH, Zhu Y, Govindasamy KM, Gopalan G. Downregulation of Aurora-A overrides estrogen-mediated growth and chemoresistance in breast cancer cells. Endocr Relat Cancer. 2008; 15:765–775.

22. Drury SC, Detre S, Leary A, Salter J, Reis-Filho J, Barbashina V, Marchio C, Lopez-Knowles E, Ghazoui Z, Habben K, Arbogast S, Johnston S, Dowsett M. Changes in breast cancer biomarkers in the IGF1R/PI3K pathway in recurrent breast cancer after tamoxifen treatment. Endocr Relat Cancer. 2011; 18:565–577.

23. Kinyamu HK, Archer TK. Estrogen receptor-dependent proteasomal degradation of the glucocorticoid receptor is coupled to an increase in mdm2 protein expression. Mol Cell Biol. 2003; 23:5867–5881.

24. Ivanova MM, Luken KH, Zimmer AS, Lenzo FL, Smith RJ, Arteel MW, Kollenberg TJ, Mattingly KA, Klinge CM. Tamoxifen increases nuclear respiratory factor 1 transcription by activating estrogen receptor beta and AP-1 recruitment to adjacent promoter binding sites. FASEB J. 2011; 25:1402–1416.

25. Johansson HJ, Sanchez BC, Mundt F, Forshed J, Kovacs A, Panizza E, Hultin-Rosenberg L, Lundgren B, Martens U, Mathe G, Yakhini Z, Helou K, Krawiec K, Kanter L, Hjerpe A, Stal O, et al. Retinoic acid receptor alpha is associated with tamoxifen resistance in breast cancer. Nat Commun. 2013; 4:2175.

26. Chiarenza A, Lazarovici P, Lempereur L, Cantarella G, Bianchi A, Bernardini R. Tamoxifen inhibits nerve growth factor-induced proliferation of the human breast cancerous cell line MCF-7. Cancer Res. 2001; 61:3002–3008.

27. Koutras AK, Fountzilas G, Kalogeras KT, Starakis I, Iconomou G, Kalofonos HP. The upgraded role of HER3 and HER4 receptors in breast cancer. Crit Rev Oncol Hematol. 2010; 74:73–78.

28. Mehta A, Tripathy D. Co-targeting estrogen receptor and HER2 pathways in breast cancer. Breast. 2014; 23:2–9.

29. Moerkens M, Zhang Y, Wester L, van de Water B, Meerman JH. Epidermal growth factor receptor signalling in human breast cancer cells operates parallel to estrogen receptor alpha signalling and results in tamoxifen insensitive proliferation. BMC Cancer. 2014; 14:283.

30. Yumoto N, Yu X, Hatakeyama M. Expression of the ErbB4 receptor causes reversal regulation of PP2A in the Shc signal transduction pathway in human cancer cells. Mol Cell Biochem. 2006; 285:165–171.

31. Neubrand VE, Cesca F, Benfenati F, Schiavo G. Kidins220/ARMS as a functional mediator of multiple receptor signalling pathways. J Cell Sci. 2012; 125:1845–1854.

32. Zheng A, Kallio A, Harkonen P. Tamoxifen-induced rapid death of MCF-7 breast cancer cells is mediated via extracellularly signal-regulated kinase signaling and can be abrogated by estrogen. Endocrinology. 2007; 148:2764–2777.

33. Browne BC, Hochgrafe F, Wu J, Millar EK, Barraclough J, Stone A, McCloy RA, Lee CS, Roberts C, Ali NA, Boulghourjian A, Schmich F, Linding R, Farrow L, Gee JM, Nicholson RI, et al. Global characterization of signalling

networks associated with tamoxifen resistance in breast cancer. FEBS J. 2013; 280:5237–5257.

34. dos Santos AR, Lopes-Costa PV, Gontijo JA, da Silva BB. Effect of tamoxifen on extracellular signal-regulated kinases in the urethra of castrated female rats. Eur J Obstet Gynecol Reprod Biol. 2012; 164:102–104.

35. Tomlinson DC, Knowles MA, Speirs V. Mechanisms of FGFR3 actions in endocrine resistant breast cancer. Int J Cancer. 2012; 130:2857–2866.

36. Turner N, Pearson A, Sharpe R, Lambros M, Geyer F, Lopez-Garcia MA, Natrajan R, Marchio C, Iorns E, Mackay A, Gillett C, Grigoriadis A, Tutt A, Reis-Filho JS, Ashworth A. FGFR1 amplification drives endocrine therapy resistance and is a therapeutic target in breast cancer. Cancer Res. 2010; 70:2085–2094.

37. Cesarone G, Garofalo C, Abrams MT, Igoucheva O, Alexeev V, Yoon K, Surmacz E, Wickstrom E. RNAi-mediated silencing of insulin receptor substrate 1 (IRS-1) enhances tamoxifen-induced cell death in MCF-7 breast cancer cells. J Cell Biochem. 2006; 98:440–450.

38. McGlynn LM, Kirkegaard T, Edwards J, Tovey S, Cameron D, Twelves C, Bartlett JM, Cooke TG. Ras/Raf-1/MAPK pathway mediates response to tamoxifen but not chemotherapy in breast cancer patients. Clin Cancer Res. 2009; 15:1487–1495.

39. Frackelton AR, Jr., Lu L, Davol PA, Bagdasaryan R, Hafer LJ, Sgroi DC. p66 Shc and tyrosine-phosphorylated Shc in primary breast tumors identify patients likely to relapse despite tamoxifen therapy. Breast Cancer Res. 2006; 8:R73.
